# Two 1D homochiral heterometallic chains: crystal structures, spectra, ferroelectricity and ferromagnetic properties†

**DOI:** 10.1039/d0ra00732c

**Published:** 2020-02-14

**Authors:** Zhuoqiang Zhou, Ming-Xing Li, Yan Sui, Emmanuel N. Nfor, Zhao-Xi Wang

**Affiliations:** Department of Pharmaceutical Engineering, College of Materials & Energy, South China Agricultural University Guangzhou 510642 PR China; Department of Chemistry, Centre for Supramolecular Chemistry and Catalysis, Shanghai University Shanghai 200444 PR China zxwang@shu.edu.cn; School of Chemistry and Chemical Engineering, The Key Laboratory of Coordination Chemistry of Jiangxi Province, Jinggangshan University Ji'an Jiangxi 343009 PR China suiyan@jgsu.edu.cn; Department of Chemistry, Faculty of Science, University of Buea POBox 63 Buea Cameroon

## Abstract

Two new homo chiral Cu–Ln (Ln = Gd and Ho) compounds bearing a chiral Schiff base ligand (1*R*,3*S*)-*N*′,*N*′′-bis[3-methoxysalicylidene]-1,3-diamino-1,2,2-trimethylcyclopentane (H_2_L) have been synthesized and characterized by elemental analysis, IR spectroscopic and single-crystal X-ray diffraction techniques. The compounds were found to exhibit 1D zig-zag skeletons with double μ-1,5 bridging dicyanamide anions. Circular dichroism (CD) spectra have been used to verify their chiroptical activities. Magnetic studies suggest that 1 and 2 hold the same magnetic behavior with the dinuclear compounds presenting ferromagnetic interaction. Furthermore, both compounds show ferroelectricity with the remnant polarization (*P*_r_) value of 0.23 and 0.18 μC cm^−2^ at room temperature, respectively.

## Abstract 2

Two homochiral 1D heterometallic chains are potential multifunctional molecules coexisting optical activity, ferromagnetic and ferroelectric properties.

## Graphical abstract

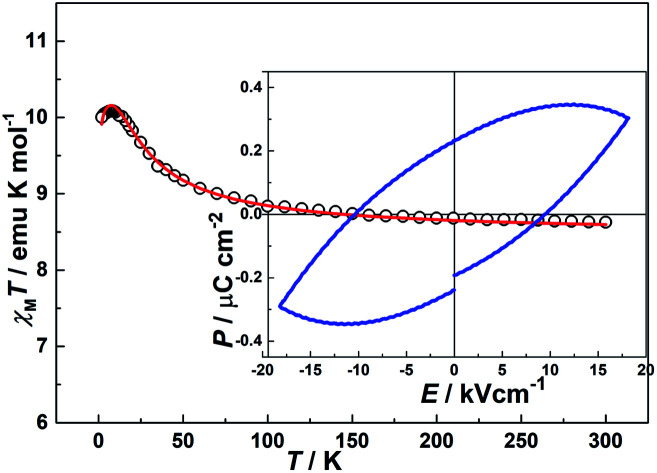

## Introduction

The rational design and synthesis of heterometallic 3d–4f compounds have attracted wide spread attention in the field of coordination chemistry owing to their fascinating architectures and potential applications in luminescence, catalysis, and molecular magnetism.^1–3^ In molecular magnetism, the intermediate magnetic coupling between 3d–4f ions may effectively suppress the zero-field quantum tunneling mechanism (QTM) thereby improving the energy barrier for spin reversal.^4^ Thus, single-molecule magnets (SMMs) with large energy barriers can be easily obtained by combining paramagnetic 3d-metal ions with highly anisotropic lanthanide ions,^5^ as in the case of [Mn_6_^III^O_3_(saO)_6_(OCH_3_)_6_Tb_2_(CH_3_OH)_4_(H_2_O)_2_] (saOH_2_ = salicylaldoxime) and [Dy_2_Co_2_(L)_4_(NO_3_)_2_(DMF)_2_]·2DMF (H_2_L = (*E*)-2-ethoxy-6-(((2-hydroxyphenyl)imino)methyl)phenol) with energy gaps of 103 K and 125 K, respectively.^6^ On the other hand, considering the ferromagnetic exchange interaction between Cu^II^ and Ln^III^ ions, their compounds (Cu–Gd) have generated special interest in magnetic refrigerants.^7^ Furthermore, much higher effective energy barriers and blocking temperatures have been achieved in lanthanide-containing SMMs as compared to most discovered transition metal-based SMMs.^3^ There are several reports on multitudinous molecular structures of 3d–4f heterometallic compounds with attractive topologies and multifarious interesting magnetic properties to date,^8^ with relatively few studies devoted to the exploration of multifunctional 3d–4f compounds combining ferroelectricity, optical activity, and ferromagnetic properties.^9^

**Scheme 1:**
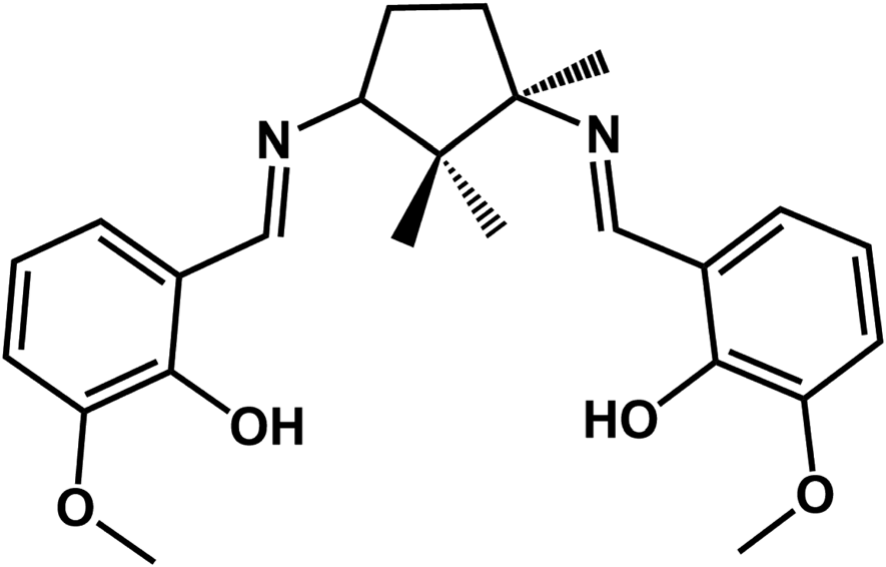
Chemical structure of H_2_L.

It is generally accepted that chiral metal coordination compounds with large net dipole moments might possess desirable ferroelectric properties, when crystallized in polar space groups.^10^ In order to synthesize optically active compounds, researchers have widely use chiral Schiff-base ligands to transfer chiral information to the assemblies.^11^ In our previous works, we have reported on a series of copper compounds with chiral Schiff-base ligands derived from camphoric diamine and different aromatic aldehydes which revealed that homochirality can be kept in 1D chain and 2D network by changing the substituent groups on aromatic rings of aldehydes.^12,13^ In continuation of our studies on homochiral coordination polymers based on camphoric diamine Schiff-base ligands, we strive to exploit new multifunctional molecular-based materials exhibiting optical activity, magnetism and ferroelectricity in one molecule. In the present work, we report on two novel homochiral 1D heterometallic Cu–Ln coordination polymers namely, [LCuLn(dca)_2_(NO_3_)]_*n*_ [dca = dicyanamide anion, Ln = Gd (1) and Ho (2)] using a new chiral Schiff base ligand (1*R*,3*S*)-*N*′,*N*′′-bis[3-methoxysalicylidene]-1,3-diamino-1,2,2-trimethyl-cyclopentane (H_2_L) (Scheme 1) and a dicyanamide anion linkage exhibiting optical activity, ferroelectricity and ferromagnetic properties.

## Experimental

### Materials and methods

All chemicals used were of analytical grade and obtained from commercial sources without further purification. Schiff base ligand H_2_L was prepared according to the literature methods using d-(+)-camphor as the starting materials.^14^ Building blocks [LCuLn(NO_3_)_3_]·Me_2_CO were prepared according to the literature methods.^15^ FT-IR spectra were recorded on a Nicolet Avatar A370 spectrometer using KBr pellets in the 400–4000 cm^−1^ region. Elemental analyses for carbon, hydrogen and nitrogen were carried out on a Vario EL III elemental analyzer. UV-vis spectra were performed with a Puxi TU-1900 spectrometer with a 1.0 cm quartz cell in DMSO solvent. Circular dichroism spectra were measured with a JASCO J-815 spectro polarimeter using the same solutions as those for the UV-vis determination. The *P*–*E* hysteresis loops of 1 and 2 were measured on a Ferroelectric Tester Precision Premier II made by Radiant Technologies Inc. at room temperature based on the compressed powder sample. Variable-temperature magnetic susceptibility measurements were taken at an applied field of 100 Oe on a Quantum Design MPMS-XL7 SQUID magnetometer working in the temperature range of 300–1.8 K. The molar magnetic susceptibilities were corrected for the diamagnetism estimated from Pascal's tables and for sample holder by previous calibration.

### Synthetic procedures

#### Synthesis of [LCuLn(dca)_2_(NO_3_)]_*n*_ [Ln = Gd (1) and Ho (2)]

A similar procedure was adopted to prepare compounds 1 and 2.

#### [LCuGd(dca)_2_(NO_3_)]_*n*_ (1)

Na(dca) (0.7 mmol, 62.4 mg) solid was slowly added to a 10 mL methanolic solution [LCuGd(NO_3_)_3_]·Me_2_CO (0.1 mmol, 87.3 mg) with continuous stirring for 30 min. The mixture was then filtered and kept at room temperature undisturbed for slow evaporation. After two days, green crystals of 1 were obtained and washed with ether. The yield was about 42% based on Gd(iii). Elemental analysis calcd (%) for C_28_H_28_CuGdN_9_O_7_: C, 40.81; H, 3.401; N, 15.30. Found: C, 40.42; H, 3.388; N, 15.28. Selected IR data (KBr, cm^−1^): 3440m, 3065w, 2977w, 2294s, 2226s, 2171s, 1616s, 1564m, 1478s, 1351m, 1296s, 1246s, 1223s, 1172w, 1081w, 975w, 854w, 784w, 743m.

#### [LCuHo(dca)_2_(NO_3_)]_*n*_ (2)

The synthetic procedure of compound 2 was similar to compound 1, except that [LCuHo(NO_3_)_3_]·Me_2_CO was used. The yield was 24% based on Ho(iii). Elemental analysis calcd (%) for C_28_H_28_CuHoN_9_O_7_: 40.43; H, 3.369; N, 15.16. Found: C, 40.38; H, 3.384; N, 15.48. Selected IR data (KBr, cm^−1^): 3440m, 3065w, 2978w, 2294s, 2225s, 2170s, 1616s, 1563m, 1477s, 1351m, 196s, 1245s, 1223s, 1172w, 1081w, 974w, 854w, 784w, 743m.

### Crystallography

The perfect crystals of compounds 1 and 2 were carefully chosen to determine the X-ray diffraction. The crystal data were collected on a Bruker Smart Apex-II CCD diffractometer at room temperature. Intensities were collected with graphite monochromatized Mo-Kα radiation (*λ* = 0.71073 Å), using the *φ* and *ω* scan technique. The data reduction was made with SAINT package. Absorption corrections were performed using SADABS program. The structures were solved by the direct methods and refined on *F*^2^ by full-matrix least-squares using SHELXTL-2014 program package with anisotropic displacement parameters for all non-hydrogen atoms. Hydrogen atoms were introduced in calculations using the riding model. The crystal data and structural refinement results are summarized in Table 1.

**Table 1:** Crystallographic data and details of refinements for 1 and 2

Compounds	1 (CuGd)	2 (CuHo)
Empirical formula	C_28_H_28_CuGdN_9_O_7_	C_28_H_28_CuHoN_9_O_7_
Formula weight	823.38	831.06
Crystal system	Monoclinic	Monoclinic
Space group	*P*2_1_	*P*2_1_
*a* (Å)	9.8268(7)	9.862(3)
*b* (Å)	15.4881(11)	15.473(5)
*c* (Å)	10.5137(7)	10.493(3)
*β* (°)	101.4330(10)	101.542(4)
*V* (Å^3^)	1568.42(19)	1568.8(8)
*Z*	2	2
*ρ*_calcd_ (g cm^−3^)	1.743	1.759
*μ* (mm^−1^)	2.834	3.241
*F*(000)	816	822
GOF (*F*^2^)	1.083	1.075
Flack parameter	0.012(14)	0.015(7)
*R*_1_ a [*I* > 2*σ*(*I*)]	0.0237	0.0166
*wR*_2_ b [*I* > 2*σ*(*I*)]	0.0738	0.0460

a *R*_1_ = ∑||*F*_o_| − |*F*_c_||/∑|*F*_o_|.

b *wR*_2_ = [∑*w*(|*F*_o_^2^| − |*F*_c_^2^|)^2^/∑*w*(|*F*_o_^2^|)^2^]^1/2^.

## Results and discussion

### Synthesis

For the design of multiferroic compounds 1 and 2, the ferroelectric property in the chiral ferromagnetic Cu^II^Ln^III^ complexes, was induced by utilizing camphoric diamine Schiff base ligands. To retain the ferromagnetic properties in compounds, dicyanamide (dca) anions were employed as bridges, due to their weak magnetic exchange interaction in μ-1,5 coordination mode.^16^ Additionally, the dca anions were used to replace parts of coordinated nitrate anions for generating electric dipolar moments. With these considerations in mind, the molar ratio of [LCuGd(NO_3_)_3_]·Me_2_CO and Na(dca) were controlled in the reaction. Compounds 1 and 2 were obtained, when the metal–ligand ratios ranged from 1 : 4 to 1 : 8. When the metal–ligand ratio was greater than 1 : 9, 2D achiral compounds were obtained, as such the metal–ligand ratios of 1 : 7 were selected for synthesis of 1 and 2.

### Crystal structures of 1 and 2

**Fig. 1:**
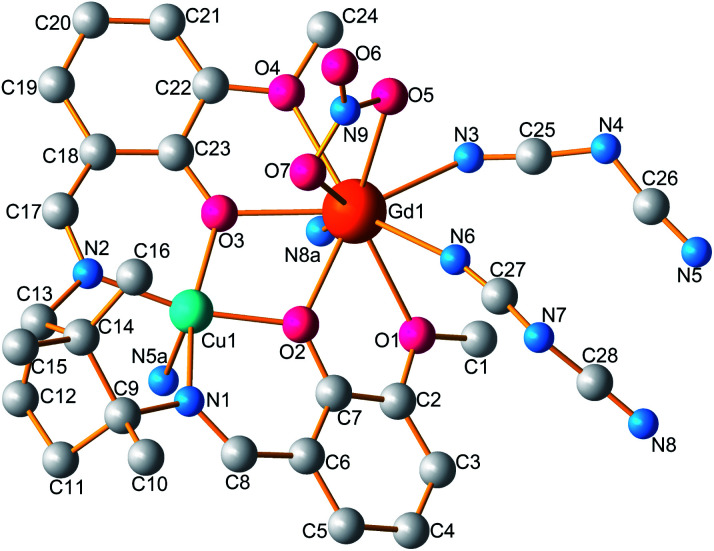
Molecular structure of 1. For clarity, hydrogen atoms are omitted. (Symmetry codes: (a) 1 − *x*, 1/2 + *y*, 2 − *z*).

The single crystal X-ray diffraction analyses revealed that compounds 1 and 2 are isomorphous and as such, only the structure of 1 is discussed in detail herein. Compound 1 crystallizes in the monoclinic *P*2_1_ space group and consists of a 1D infinite chain. As shown in Fig. 1, the asymmetric unit is composed of a dinuclear [LCuGd]^3+^ moiety, one nitrate anion, and two bridgings dca. The [LCuGd] unit preserves the structural features of the whole [CuLn] family of binuclear complexes in which Cu(ii) and Gd(iii) ions are connected by two μ_2_-phenoxo oxygen atoms (O2 and O3).^15,17^ The copper(ii) ion with a distorted square pyramidal geometry is hosted within the inner N_2_O_2_ compartment which formed the basal plane. The apical site is occupied by nitrogen atom from dca group (Cu1–N5A 2.280(8) Å, Table 2). While the Gd(iii) ion occupies the outer O_4_ cavity surrounded by six oxygen and three nitrogen atoms arising from the L^2−^ ligand, from one nitrate, and from the three dca anions, respectively. The Cu–Gd separation of 3.4884(9) Å is same as that in previously reported works.^18^ The two Gd–O bonds involving the –OMe side arms (2.591(5) and 2.518(6) Å) are longer than the ones from the phenolate oxygens (2.305(4) and 2.369(6) Å). The two Gd–O(nitrate) bonds have lengths of 2.518(7) and 2.510(7) Å, whereas the Gd–N bond lengths vary from of 2.436(7) to 2.485(8) Å.

**Table 2:** Selected bond distances (Å) and bond angles (°) for 1 and 2^a^

1		2	
Cu1–N1	2.047(7)	Cu1–N1	2.040(5)
Cu1–N2	1.962(7)	Cu1–N2	1.964(5)
Cu1–N5A	2.280(8)	Cu1–N5A	2.295(6)
Cu1–O2	1.946(7)	Cu1–O2	1.946(5)
Cu1–O3	1.976(5)	Cu1–O3	1.970(4)
Gd1–N3	2.436(7)	Ho1–N3	2.399(5)
Gd1–N6	2.485(8)	Ho1–N6	2.465(6)
Gd1–N8A	2.461(8)	Ho1–N8A	2.436(6)
Gd1–O1	2.591(5)	Ho1–O1	2.578(4)
Gd1–O2	2.305(4)	Ho1–O2	2.272(3)
Gd1–O3	2.369(6)	Ho1–O3	2.340(4)
Gd1–O4	2.518(6)	Ho1–O4	2.506(4)
Gd1–O5	2.518(7)	Ho1–O5	2.483(5)
Gd1–O7	2.510(7)	Ho1–O7	2.479(5)
Cu1–Gd1	3.4884(9)	Cu1–Ho1	3.4538(10)
Cu1–O2–Gd1	110.0(2)	Cu1–O2–Ho1	109.7(2)
Cu1–O3–Gd1	106.5(2)	Cu1–O3–Ho1	106.19(18)

a Symmetry codes: (A) 1 − *x*, 1/2 + *y*, 2 − *z*.

**Fig. 2:**
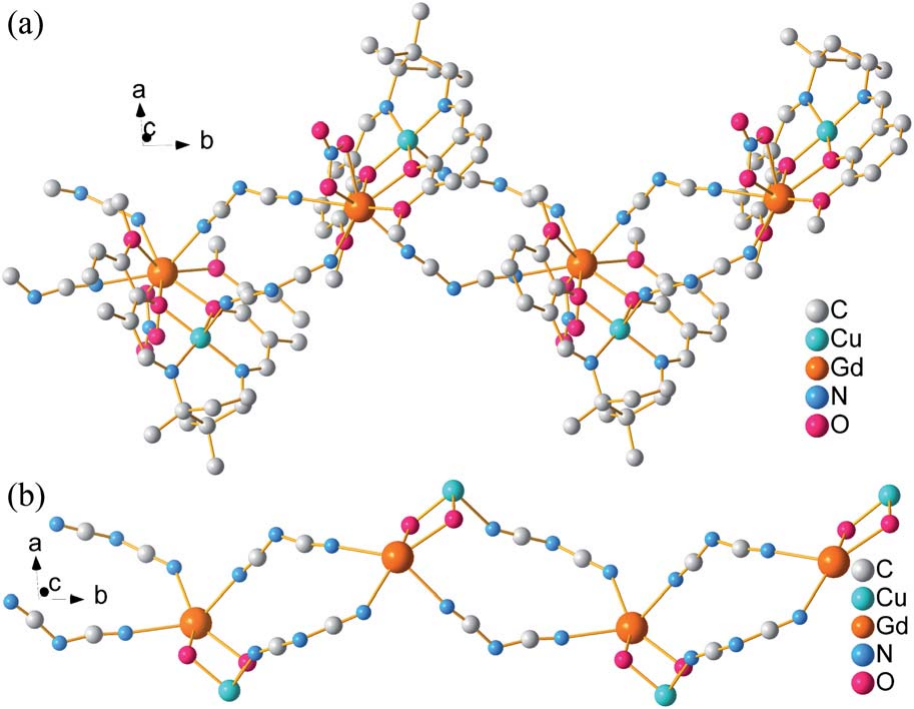
Zig-zag chain (a) and skeleton (b) of 1 along *b*-axis. Hydrogen atoms are deleted for clarity.

Each [LCuGd(NO_3_)]^2+^ unit connects other symmetry-related dinuclear centres by two dca groups with μ-1,5 bridge mode, forming a 1D infinite zig-zag chain along *b*-axis (Fig. 2). In the chain, the neighbouring [LCuGd(NO_3_)]^2+^ separations are 8.7690(5), and 7.8696(4) Å for Gd⋯Gd, and Gd⋯Cu, respectively. While, the nitrate anions act as a chelating bidentate mode with one terminal oxygen atom toward outside of the chain. Adjacent chains are expended into a 2D layer through weak hydrogen bonds (Fig. S1, ESI†). Furthermore, the 2D layer is linked each other to form a 3D supramolecular framework by weak interaction (Fig. S2, ESI†).

### Spectra characterizations

The infrared spectra of 1 and 2 (Fig. S3, ESI†) are both in accordance with their structural characteristics as revealed by single-crystal X-ray diffraction. Three strong peaks of dicyanamide ligand at 2294, 2226, and 2171 cm^−1^ are observed in compound 1, corresponding to the *ν*_as_ + *ν*_s_ combination modes, *ν*_as_ and *ν*_s_ of –C≡N fragments, respectively. The spectra bands displaying at 3065, 1616 and 784 cm^−1^ are respectively assigned to *ν*(C–H), *ν*(C=C) stretching vibrations and *δ*(C–H) out-of-plane bending vibration of phenyl groups.^19^ Whereas, weak *ν*(C–H) stretching vibrations of methyl groups appear near 2977 cm^−1^. The band at 1564 cm^−1^ is designated to the –C=N stretching vibration of the Schiff base ligand. In addition, there are four absorptions of coordinated nitrate in 1478 (*ν*_4_), 1296 (*ν*_1_), 975 (*ν*_2_), and 854 cm^−1^ (*ν*_3_). The peak near 1296 cm^−1^ is the superposition of bands of phenoxy and nitrate. The difference between *ν*_1_ and *ν*_4_ is about 200 cm^−1^ (182 cm^−1^), which indicates that nitrate adopted a chelating bidentate coordination mode,^20^ in agreement with the crystal structure of 1.

**Fig. 3:**
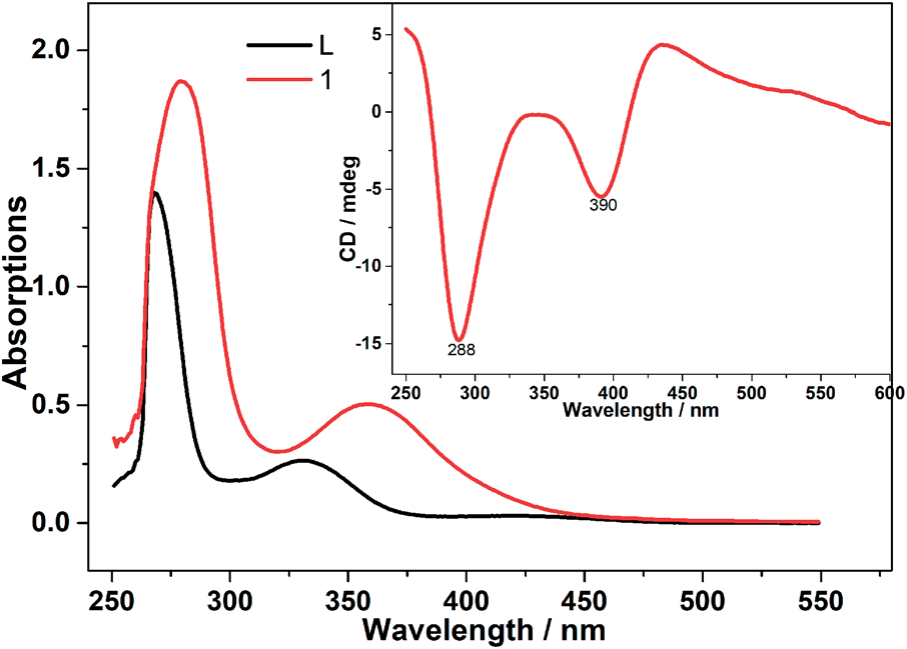
UV-vis spectra of the Schiff-base ligands (H_2_L) and compound 1 (4.93 × 10^−4^ mM) in DMSO solutions together with the corresponding CD spectra (the inset).

The UV-vis spectra of free ligand H_2_L and compound 1 in DMSO solutions are shown in Fig. 3. The sharp band at 267 cm^−1^ of L^2−^ is attributed to aromatic π–π* intraligand charge transfer transition which are shifted down to 280 cm^−1^ in 1. Another typical band appeared at 329 cm^−1^ considered as L → M charge transfer transition band which is increased to 358 cm^−1^ due to metal-ion complexation.^21^ To certify the optical activity of 1, circular dichroism (CD) spectra were carried out in DMSO solution. In the CD spectra (the inset in Fig. 3), negative Cotton effects at 288 and 390 cm^−1^ have been observed in the UV-vis region, which is a good agreement between the CD and UV-vis spectra. The CD spectra confirm the homochirality of 1.^22^

### Magnetic properties

**Fig. 4:**
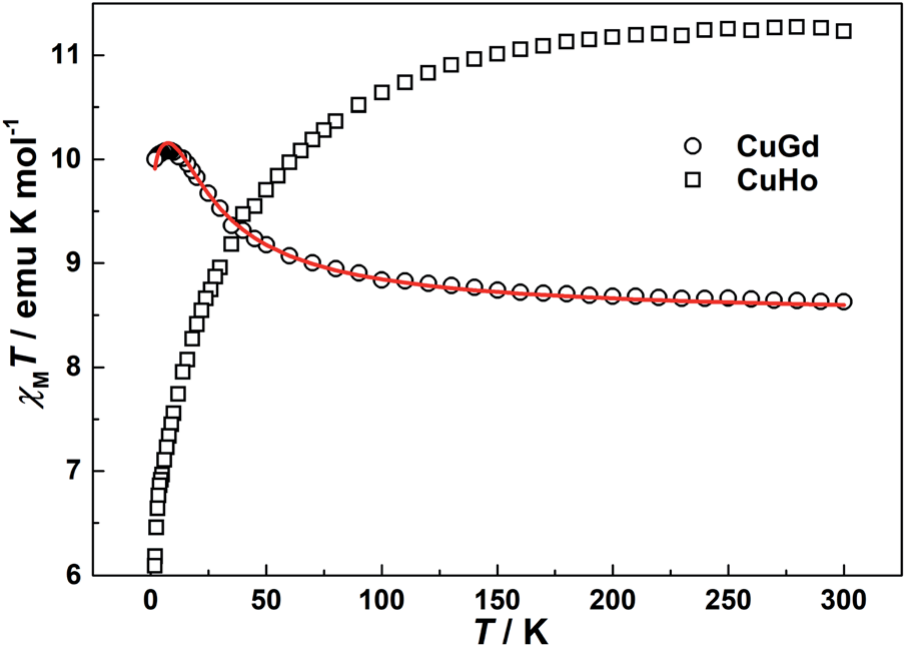
Temperature dependence of magnetic susceptibilities of 1 (CuGd) and 2 (CuHo) under an applied field of 100 Oe. Solid line represents the best fit of the data.

Temperature-dependence molar susceptibility measurements of the crystalline sample of 1 and 2 were carried out on a Quantum Design MPMS-XL7 SQUID magnetometer in an applied magnetic field of 100 Oe over the temperature range 1.8–300 K. Plots of *χ*_M_*T versus T* for 1 and 2 are depicted in Fig. 4. At room temperature, the *χ*_M_*T* value of 1 is 8.62 emu K mol^−1^, which is slightly higher than the calculated spin-only value of 8.25 emu K mol^−1^ based on uncoupled one copper(ii) and one gadolinium(iii) cations (assuming *S*_Cu(II)_ = 1/2, *S*_Gd(III)_ = 7/2, and *g* = 2.0). With the system cooling, the *χ*_M_*T* product steadily increases until it reaches a maximum value of 10.09 emu K mol^−1^ at 8 K. This magnetic behaviour indicates a ferromagnetic coupling between copper(ii) and gadolinium(iii) metal centers through double μ_2_-phenoxo oxygen bridges.^15,17^ On further lowering of the temperature, the *χ*_M_*T* plot undergoes a slightly decrease with a value of 10.00 emu K mol^−1^ at 1.8 K closed to the expected value (10 emu K mol^−1^) for total spin state *S*_T_ = 4, which may be due to magnetic saturation and/or crystal field splitting of the Gd^III^ ion.^23^ For 2, the *χ*_M_*T* value is 11.26 emu K mol^−1^, which is lower than the theoretical value of 14.42 emu K mol^−1^ for one Cu^II^ and one Ho^III^ ions. As the temperatures decrease, the *χ*_M_*T* slowly decreases down to the minimum values of 6.09 emu K mol^−1^ at 1.8 K, which may be caused by antiferromagnetic couplings between the Cu^II^ and Ho^III^ ions and/or the spin–orbit couplings of the Ho^III^ ions. The magnetic behaviour of 2 is same as those of previously reported Cu–Ho dinuclear complexes with other Schiff-base ligands.^24^

In the two compounds, the dca anion adopts bidentate μ-1,5 bridging mode, which is the most frequently observed coordination mode. Because of its long five-atomic exchange pathway, the μ-1,5 bridging dca anion exhibits only weak magnetic exchange interactions and cooperative phenomena are not observed in any of these compounds in documents.^16^ Thus, the magnetic properties of 1 and 2 can be considered as dinuclear units. Based on the isotropic Hamiltonian *Ĥ* = −2*JŜ*_Cu_·*Ŝ*_Gd_, where *J* is the coupling constants mediated by phenoxo in the dinuclear, the experimental data of 1 in the whole temperature range were fitted to the Bleaney–Bowers equation modified by Kahn and co-workers.^25^ The expression is written as follows when the mean-field corrections *zj*′ is considered:
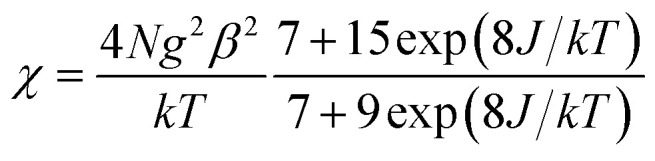
*χ*_M_ = *χ*/[1 − (2*zj*′/*Ng*^2^*β*^2^)*χ*]

Using this rough model, the magnetic susceptibilities of 1 was simulated, giving the best fit with parameters *g* = 2.026(1), *J* = 3.39(9) cm^−1^, *zj*′ = −0.0034(2) cm^−1^, with *R* = ∑[(*χ*_M_*T*)_calc_ − (*χ*_M_*T*)_obs_]^2^/∑(*χ*_M_*T*)_obs_^2^ = 1.5 × 10^−3^. The results indicate the ferromagnetic coupling between metal ions mediated double μ_2_-phenoxo oxygen bridges. The value of *J* is comparable with those reported previously in dinuclear Cu^II^–Gd^III^ systems.^15,17^

### Ferroelectric properties

**Fig. 5:**
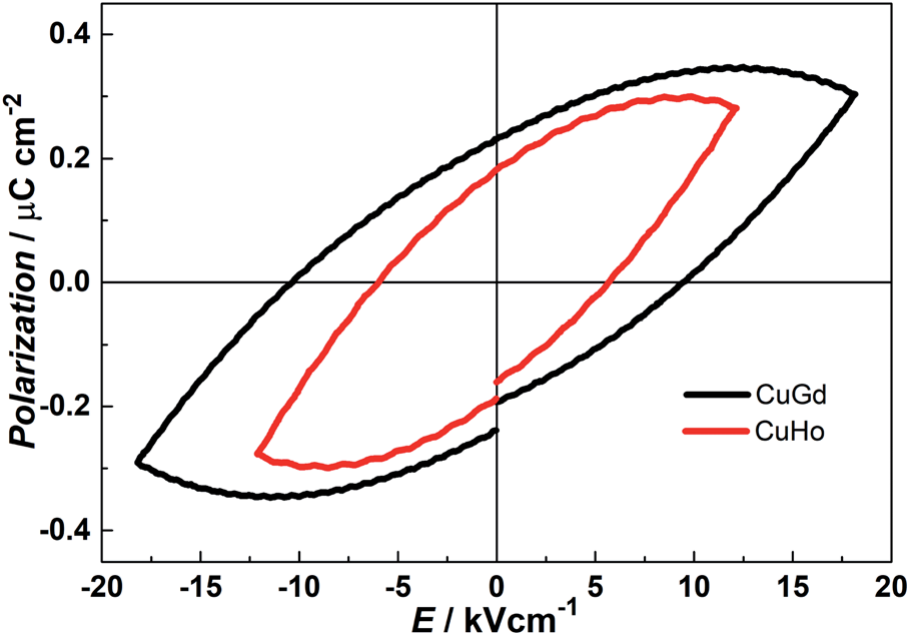
*P*–*E* hysteresis loops of 1 (CuGd) and 2 (CuHo) based on a compressed powder sample at room temperature.

It is well known that compounds crystallizing in the polar point groups may display ferroelectric behaviour.^26^ Compounds 1 and 2 crystallize in the chiral space group *P*2_1_ belonging to *C*2 polar point group, which meets the basic requirement of ferroelectric properties. The ferroelectric data of 1 and 2 were collected at room temperature with powder pellet samples. As shown in Fig. 5, both compounds exhibit well-shaped *P*–*E* hysteresis loop which is the ferroelectric features. For 1, the remnant polarization (*P*_r_) value is about 0.23 μC cm^−2^ at an applied electric field of 18.18 kV cm^−1^ with a coercitive field (*E*_c_) value of about 9.50 kV cm^−1^. And the saturation value of spontaneous polarization (*P*_S_) is about 0.35 μC cm^−2^. Whilst, we obtained *P*_r_ = 0.18 μC cm^−2^, *E*_c_ = 5.75 kV cm^−1^, and *P*_S_ = 0.30 μC cm^−2^ for 2. By comparison with those of other recently reported molecular ferroelectrics, the *P*_S_ of 1 and 2 are mediocre,^27^ but slightly larger than that of classical organic–inorganic ferroelectrics NaKC_4_H_4_O_6_·4H_2_O (*P*_S_ = 0.25 μC cm^−2^). According to the structures of 1 and 2, the ferroelectricity may originate from the off-centering of charges between [LCuLn]^3+^ dinuclear and nitrate anion, which leads to spontaneous electric dipolar moments occurring. Under an applied electric field, these dipolar moments are arranged in the same direction, which results in the appearance of ferroelectric behaviour in 1 and 2.

## Conclusions

In conclusion, we have designed, synthesized, and successfully characterized two new one-dimensional homochiral Cu–Ln (Ln = Gd and Ho) compounds bearing a chiral Schiff base ligand. Both the compounds exhibit ferroelectric and magnetic properties indicating that, the homochiral 1 and 2 are potential molecule-based multifunctional materials coexisting optical activity, ferromagnetic and ferroelectric properties in one molecule. With this synthetic strategy, molecule-based materials with fascinating multifunctionality can be obtained conveniently.

## Conflicts of interest

There are no conflicts to declare.

## Supplementary Material

RA-010-D0RA00732C-s001

RA-010-D0RA00732C-s002
