# Supplementary material for: Two 1D homochiral heterometallic chains: crystal structures, spectra, ferroelectricity and ferromagnetic properties
Source: RSC Adv. 2020 Feb 14;10(12):7004–10. doi: 10.1039/d0ra00732c (PMC9049737; doi:10.1039/d0ra00732c)
Supplement: RA-010-D0RA00732C-s001 [file RA-010-D0RA00732C-s001.pdf]

Electronic Supplementary Material (ESI) for RSC Advances.
This journal is © The Royal Society of Chemistry 2020

Electronic supplementary information

Two 1D homochiral heterometallic chains: crystal structures, spectra, ferroelectricity and ferromagnetic properties

Zhuoqiang Zhou,[a] *Ming-Xing Li,*[b] *Yan Sui,*[*c] *Emmanuel N. Nfor*[d] *and Zhao-Xi Wang*[*b]

[a] Department of Pharmaceutical Engineering, College of Materials & Energy, South China of Agricultural University, Guangzhou 510642, PR China

[b] Department of Chemistry, Centre for Supramolecular Chemistry and Catalysis, Innovative Drug Research Centre, Shanghai University, Shanghai 200444, PR China.

[c] School of Chemistry and Chemical Engineering, The Key Laboratory of Coordination Chemistry of Jiangxi Province, Jinggangshan University, Ji'an, Jiangxi 343009, PR China

[d] Department of Chemistry, University of Buea, P.O. Box 63, Buea, SWR, Cameroon

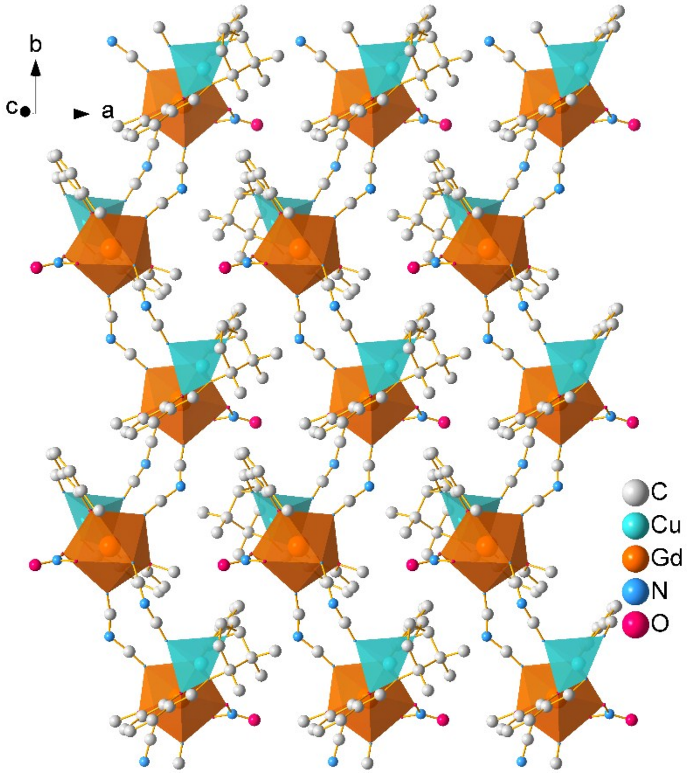

Fig.S1 2D layer of **1** in the *ab* plane.

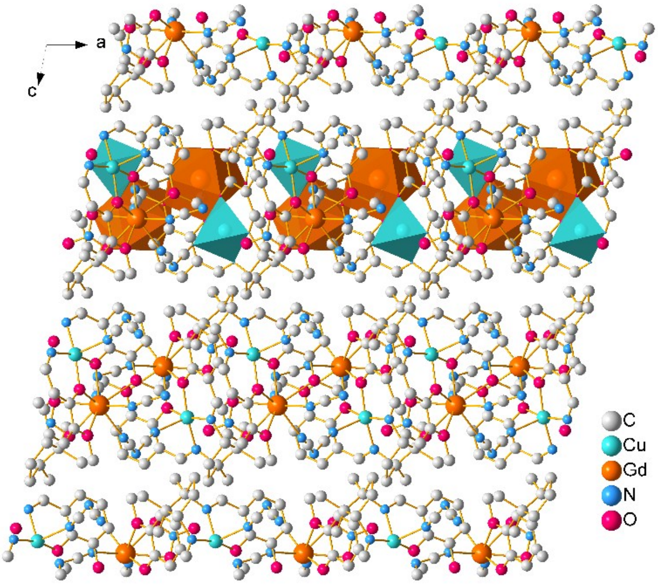

Fig.S2 3D supramolecular framework of **1**.

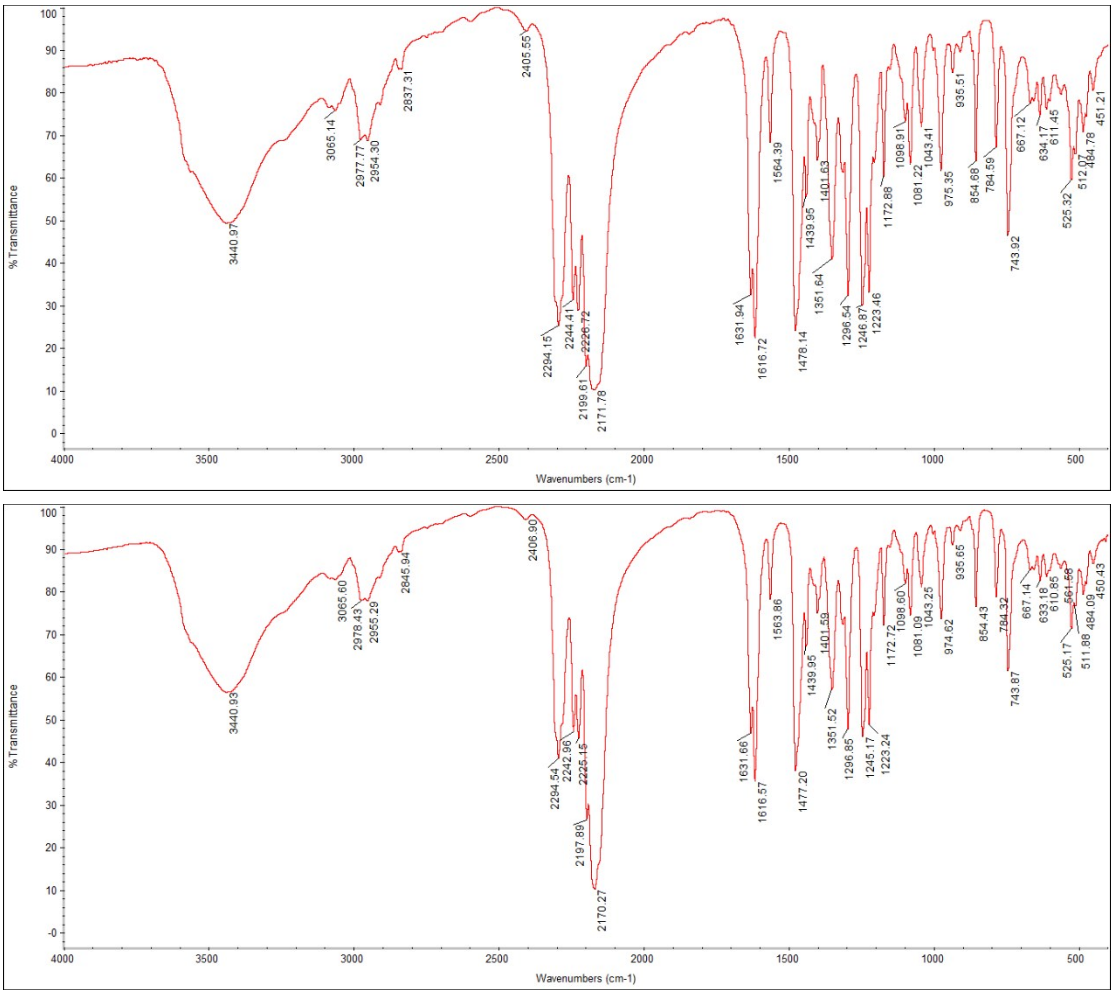

Fig.S3 Infrared spectra of **1** (up) and **2** (down).
